# Energetic plasticizers for GAP-based formulations with ADN: compatibility and performance evaluation of a nitrofurazanyl ether

**DOI:** 10.1039/d5ra05154a

**Published:** 2025-10-06

**Authors:** Patrick Lieber, Uwe Schaller, Thomas M. Klapötke

**Affiliations:** a Fraunhofer Institute for Chemical Technology Joseph-von-Fraunhofer-Str. 7 76327 Pfinztal Germany patrick.lieber@ict.fraunhofer.de; b Department of Chemistry, Ludwig-Maximilian-University of Munich Butenandtstr. 5-13 81377 Munich Germany

## Abstract

Glycidyl azide polymer (GAP) diol is an energetic binder with azide-functionalized side chains that offer a high enthalpy of formation. When formulated with ammonium dinitramide (ADN), GAP-based systems exhibit promising energetic and ballistic performance. However, GAP suffers from poor mechanical properties compared to inert binders such as hydroxyl-terminated polybutadiene (HTPB). As a result, plasticization is essential to lower the glass transition temperature of GAP. Energetic plasticizers can enhance both mechanical and energetic performance. In this study, we evaluated the compatibility of a novel nitrofurazanyl ether-based energetic plasticizer, NFPEG3N3, with key formulation components: GAP diol, HMX, Desmodur N100, and ADN. Compatibility was assessed using heat-flow microcalorimetry (HFMC) and thermogravimetry (TG), in accordance with STANAG 4147. NFPEG3N3 was found to be compatible with GAP diol, HMX, and N100. Although the NFPEG3N3–ADN mixture passed the HFMC (remaining below the 1% heat of explosion threshold), it failed TG, indicating potential concerns with long-term thermal stability. Performance calculations showed that replacing 15 wt% of GAP with NFPEG3N3 in a composite propellant increased the volume-specific impulse by 77 N s dm^−3^. Additionally, formulations incorporating NFPEG3N3 demonstrated a superior oxygen balance and higher volume-specific impulse compared to those using the widely adopted energetic plasticizer Bu-NENA.

## Abstract 2

NFPEG3N3, a nitrofurazanyl ether plasticizer, is compatible with GAP, HMX and N100 and likely incompatible with ADN; replacing 15 wt% GAP raises volume-specific impulse by 77 N s dm^−3^ and improves oxygen balance *versus* Bu-NENA.

## Graphical abstract

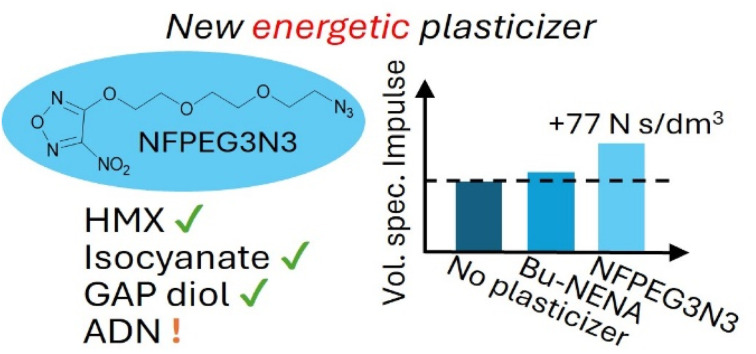

## Introduction

**Fig. 1:**
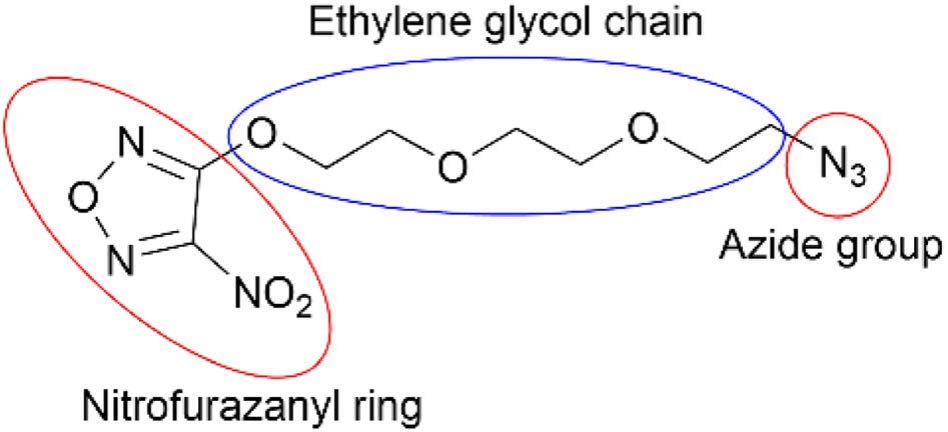
Molecular structure of NFPEG3N3 (3-(2-(2-(2-azidoethoxy)ethoxy) ethoxy)-4-nitro-1,2,5-oxadiazole).

**Fig. 2:**
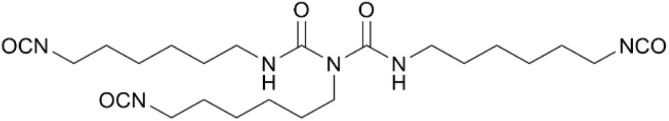
Molecular structure of HDI-biuret (1,3-bis (6-isocyanatohexyl)-1-(6-isocyanatohexylcarbamoyl)urea).

The use of energetic plasticizers is key to the performance of advanced solid propellants and polymer-bonded explosives.^1–3^ Plasticizers reduce the glass transition temperature (*T*_g_) of the polymer binder, thereby improving mechanical properties at low temperatures.^4^ Unlike inert plasticizers, energetic plasticizers incorporate explosophoric groups, such as nitrate esters, nitramines, azido or nitro functionalities, which contribute to the energetic output of a formulation. Representative energetic plasticizers are shown in the SI (S1).^5,6^ These compounds typically feature a saturated, nearly linear carbon backbone with energetic side groups.^7^ Ethylene glycol-based backbones are also common; for example, the thermal properties of BATEG highlight the strong influence of ethylene glycol segments on *T*_g_, which occurs at the remarkably low temperature of −110 °C. However, BATEG suffers from high volatility.^6,8,9^ While heterocyclic building blocks have been widely explored in energetic materials, their use as energetic plasticizers remains underrepresented in the literature.^10–12^ Among energy-rich heterocycles, oxadiazoles are especially promising due to their favorable oxygen balance compared to triazoles or tetrazoles. Of the four oxadiazole isomers, furazan (1,2,5-oxadiazole) is of particular interest, offering the highest enthalpy of formation and sufficient chemical stability.^13–15^ Recently, a novel nitrofurazanyl ether-based plasticizer, NFPEG3N3, was synthesized and evaluated for potential use as a heterocyclic energetic plasticizer (Fig. 1). NFPEG3N3 exhibits a low *T*_g_ of −72 °C, decomposition onset temperature of 167 °C, and an enthalpy of formation of −41.7 kJ mol^−1^. Compared to conventional energetic plasticizers, NFPEG3N3 significantly lowers the *T*_g_ and viscosity of glycidyl azide polymer (GAP) diol.^16^ GAP diol is a hydroxyl-terminated polyether containing azide groups in its side chains. When combined with the environmentally friendly oxidizer ammonium dinitramide (ADN), it offers excellent ballistic properties.^17^ Despite its high density and positive enthalpy of formation, GAP exhibits poor mechanical properties compared to hydroxyl-terminated polybutadiene (HTPB), a widely used inert binder.^18^ The molecular structures of GAP and HTPB are compared in the SI (S2). In addition to favorable thermal and mechanical characteristics, compatibility with formulation components is essential for the safe and effective implementation of new energetic compounds.^19^ This is particularly important when manufacturing formulations for the first time. According to STANAG 4147, compatibility should be assessed through complementary test methods. If results remain inconclusive, alternative methods must be used.^20^ In this study, we present compatibility and performance data for NFPEG3N3 in combination with GAP diol, HMX, Desmodur N100, and ADN. Fig. 2 shows the molecular structure of HDI-biuret, the trifunctional isocyanate component of the commercial hardener N100.^21^

## Results and discussion

### Compatibility of NFPEG3N3 with formulation components

The compatibility of NFPEG3N3 with HMX, GAP diol, N100, and ADN was evaluated using dynamic thermogravimetry (TG) and heat-flow microcalorimetry (HFMC) in accordance with STANAG 4147. ADN was used as spherical prills.^22^ Images of the ADN prills used, taken with an electron microscope, are included in the SI (S3). Both methods follow a similar general procedure: a reactivity value (*R*) is calculated based on either the excess weight loss (TG) or heat generation (HFMC), reflecting interactions that occur between components in the mixture. In the dynamic TG test, the total weight losses of the binary mixture (*W*_M_) is compared with the sum of the weight losses of the individual components (*W*_M,calc_) at a selected temperature. This temperature corresponds to the lowest temperature at which a derivative peak appears in the TG curve of the mixture. If the weight loss of the mixture exceeds the calculated sum of the individual losses, it indicates potential incompatibility. The percentage of mass loss due to interactions (*R*_W_) was calculated using eqn (1).

Eqn (1) calculation of weight loss-based reactivity.1
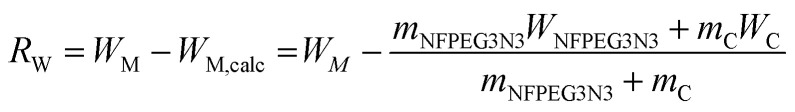


According to STANAG 4147, a weight difference of less than 4% indicates that the materials are compatible.

A difference between 4% and 20% suggests potential incompatibility and warrants further testing, while a difference greater than 20% indicates clear incompatibility. Table 1 summarizes the excess weight loss due to interactions in binary mixtures containing NFPEG3N3, along with the corresponding compatibility assessments. Mixtures of NFPEG3N3 with HMX, GAP diol, and N100 exhibited a lower weight loss than the calculated sum for the individual components, indicating compatibility. In contrast, the mixture with ADN showed an excess weight loss of 5.2%, which exceeds the 4% threshold and therefore requires further investigation. The observed negative reactivity values in some mixtures may be attributed to their elevated viscosity, influenced by the presence of high-viscosity components such as GAP diol and N100, as well as finely dispersed solid HMX. This higher viscosity appears to retard the evaporation of NFPEG3N3 compared to the single-compound tests. All TG curves are provided in the SI (S4). The compatibility between NFPEG3N3 and the formulation components was further evaluated using isothermal heat-flow microcalorimetry (HFMC). HFMC is a highly sensitive technique for measuring the heat generation rate of a sample under isothermal conditions. Because it uses larger sample sizes, typically in the gram range, HFMC provides a more representative assessment than screening methods such as TG, which typically use milligram-scale samples. As in the TG study, both pure substances and a binary mixture at a 1 : 1 weight ratio were analyzed to assess thermal interactions and determine compatibility. The expected heat release for each binary mixture (*Q*_M,calc_) was calculated from the individual measurements of the two components, as shown in eqn (2).

**Table 1:** Compatibility assessment by dynamic thermogravimetry (ff. = fulfilled)

Component	*T*_dTGA_ a [°C]	*W*_NFPEG3N3_ b [%]	*W*_C_ c [%]	*W*_M_ d [%]	*R*_W_ e [%]	Assmt.
HMX	175	95.7	0.1	37.9	−10.6	ff.
GAP diol	172	95.1	0.3	29.9	−17.5	ff.
N100	183	96.7	6.0	34.3	−19.1	ff.
ADN	158	52.3	20.1	42.1	+5.2	

a Mixture derivative peak temperature.

b Weight loss of NFPEG3N3.

c Weight loss of component.

d Weight loss of binary mixture.

e Reactivity by excess weight loss.

Eqn (2) calculation of heat flow-based reactivity.2
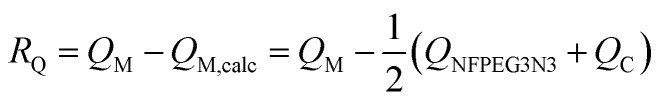


**Fig. 3:**
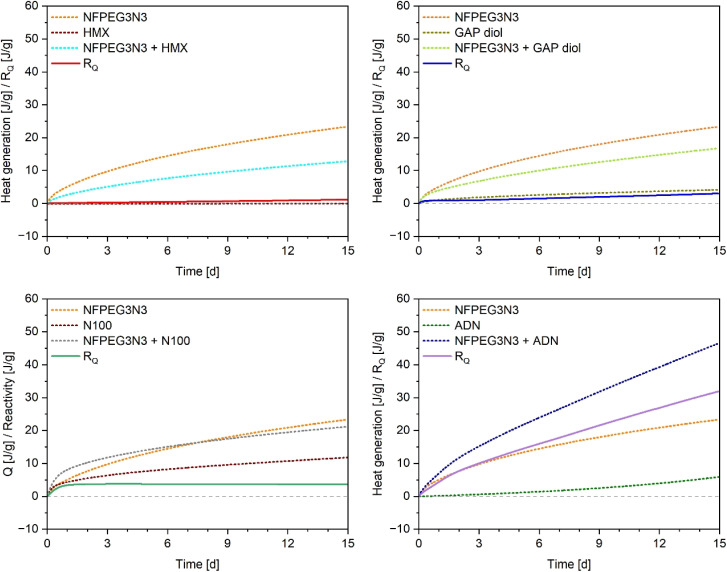
Heat generation data of components and mixtures (1 : 1 by mass) and the reactivity functions at 80 °C.

It was subtracted from the experimentally measured value of the mixture (*Q*_M_) to determine the heat generated by interactions (*R*_Q_). Fig. 3 displays the heat generation profiles of the pure components and their binary mixtures, along with the calculated excess heat generation (*R*_Q_) over the 15-day test period at a constant temperature of 80 °C. In the HFMC test, materials are considered compatible if the heat generated from reactive interactions remains below a threshold during the defined standard assessment period of 10.6 days. Measurements were extended to 15 days to ensure the detection of slow or delayed exothermic reactions.

While a general compatibility limit of 30 J g^−1^ is widely accepted for nitrocellulose (NC)-based energetic materials, no universal threshold exists for other classes of energetic compounds. More recently, a threshold of 1% of the heat of explosion of the mixture has been proposed and is now included in STANAG 4147.^19^ Detailed heat flow and reactivity data are provided in the SI (S5). Based on this criterion, NFPEG3N3 was found to be compatible with HMX and GAP diol, with heat generation well below the threshold. For the ADN, the HFMC threshold was barely met, suggesting borderline compatibility. However, when considered alongside the TG results, the overall findings indicate likely incompatibility according to STANAG 4147. One potential solution to this issue could be the use of polymer-coated ADN prills to reduce interactions between the substances.^23,24^ In the case of N100, no heat of explosion-based threshold could be calculated due to unavailable thermochemical data. However, the observed heat release was sufficiently low that, when compared with the 30 J g^−1^ limit, compatibility can be assumed. Table 2 summarizes the compatibility test results and the corresponding threshold values used in the assessment. The use of untreated ADN with NFPEG3N3 is questionable due to their likely incompatibility. However, NFPEG3N3 has been shown to be suitable for incorporation into high-performance polymer-bonded explosive (PBX) formulations containing GAP diol, N100, and HMX.

**Table 2:** Summary of compatibility test results and limit values

Compatibility test	*R*_Q_ a [J g^−1^] (limit value)^b^	*R*_W_ c [%] at *x* °C	
Threshold values	−1% of *Q*_Ex_*R*_Q_ < 1% of *Q*_Ex_	*R*_W_ < 4%	Total assessment
NFPEG3N3 + HMX	0.80 (38.42)	−10.6 at 175 °C	Compatible
NFPEG3N3 + GAP diol	2.24 (32.25)	−17.5 at 172 °C	Compatible
NFPEG3N3 + N100	3.67 (30.00^d^)	−19.1 at 183 °C	Compatible
NFPEG3N3 + ADN	24.46 (41.90)	+5.2 at 158 °C	Incompatibility is likely

a Reactivity by excess heat generation over 10.6 days at 80 °C measured with HFMC.

b Limit value of 1% of the heat of explosion of the specified mixture (1 : 1 weight ratio).

c Reactivity by excess weight loss at the specified temperature measured with dynamic TG.

d General limit value for NC-based formulations.^20^

### Impact of NFPEG3N3 on the thermodynamic performance of a composite propellant

**Fig. 4:**
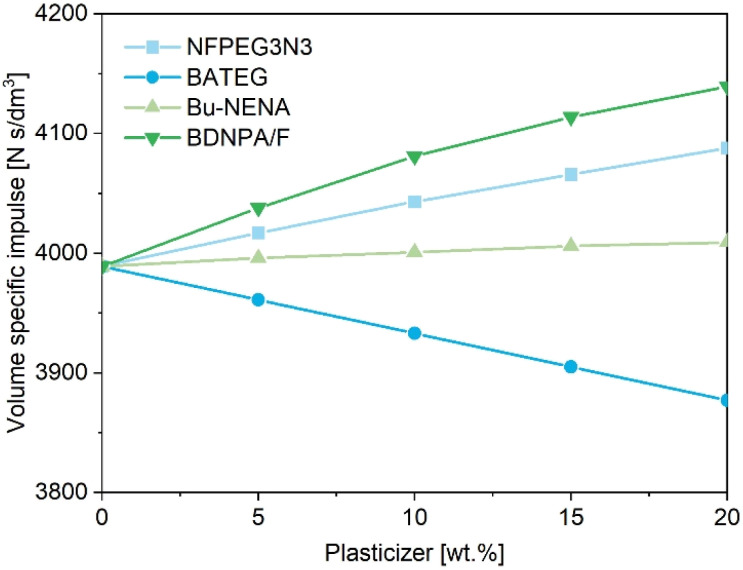
Impact of plasticizers on the volume-specific impulse of GAP-based propellants containing HMX and ADN.

The impact of NFPEG3N3 on the volume-specific impulse of a GAP-based propellant containing ADN as an environmentally friendly oxidizer was evaluated by theoretical calculations. The performance results were compared with those of commonly used energetic plasticizers BDNPA/F and Bu-NENA, as well as the ethylene glycol compound BATEG (S1). The selected formulation contained a total solid filler content of 70 wt%, consisting of 58.7 wt% ADN and 11.3 wt% HMX. The binder and plasticizer together accounted for 30 wt% of the composition. In the thermodynamic calculations, up to 20 wt% of the GAP binder was replaced by plasticizer. Fig. 4 illustrates the impact of the plasticizers on the volume-specific impulse. Replacing 15 wt% of GAP with NFPEG3N3 increased the volume-specific impulse by 77 N s dm^−3^, from 3989 N s dm^−3^ to 4066 N s dm^−3^. This represents a significant performance improvement, especially when considering that substituting the same proportion of GAP with a common inert plasticizer as dioctyl adipate (DOA) reduces the volume-specific impulse by 434 N s dm^−3^.

Among the energetic plasticizers studied, NFPEG3N3 provided a greater increase in the volume-specific impulse than BATEG and Bu-NENA. Although BDNPA/F demonstrated slightly higher performance gains in terms of volume-specific impulse, the benefits of NFPEG3N3, particularly in terms of its favorable thermal stability, remain substantial. Tabulated values for all calculated volume and mass-specific impulses are provided in the SI (S6). Table 3 provides a more detailed comparison of the thermodynamic performance of formulations containing 10 wt% of the evaluated energetic plasticizers. The formulation incorporating NFPEG3N3 exhibits a lower mass-specific impulse compared to those containing BDNPA/F or Bu-NENA, but a higher one than the formulation with BATEG. It also displays a more favorable oxygen balance than those with BATEG or Bu-NENA. Although the BDNPA/F-based formulation shows the highest overall performance parameters, previous studies have indicated that BDNPA/F is relatively ineffective at reducing the glass transition temperature and viscosity of GAP diol.^16,25,26^ Because of its greater effect on the volume-specific impulse, the formulation with NFPEG3N3 is better suited for near-surface applications. This is particularly important in scenarios where compact propulsion systems are required to minimize air resistance, and performance per unit volume is a critical design parameter.

**Table 3:** Calculated thermodynamic performance of composite propellants consisting of 58.7 wt% ADN, 11.3 wt% HMX, 20 wt% GAP and 10 wt% energetic plasticizers at an expansion ratio of 70 : 1

Plasticizer [type]	*I*_sp_ a [N s kg^−1^]/[s]	*V*_sp_ b [N s dm^−3^]	*ρ* c [g cm^−3^]	*T*_c_ d [°C]	*T*_a_ e [°C]	OB^f^ [%]
BDNPA/F	2490/253.8	4081	1.64	2984	1291	−17.3
Bu-NENA	2481/252.9	4001	1.61	2880	1218	−22.0
BATEG	2458/250.6	3933	1.15	2806	1165	−24.3
NFPEG3N3	2477/252.5	4043	1.63	2921	1241	−20.4

a Mass-specific impulse.

b Volume-specific impulse.

c Formulation density.

d Chamber temperature.

e Nozzle temperature.

f Oxygen balance.

## Conclusion

NFPEG3N3 is compatible with GAP diol, HMX, and Desmodur N100 by HFMC and TG in accordance with STANAG 4147. For N100, a heat of explosion-based threshold could not be defined, but the measured heat release was well below the widely used 30 J g^−1^ limit. The NFPEG3N3-ADN pair met the HFMC threshold set at 1% of the mixture's heat of explosion but failed dynamic TG (5.2% excess mass loss), indicating likely long-term thermal incompatibility with untreated ADN. Thermodynamic calculations for a representative ADN/HMX/GAP propellant show that replacing 15 wt% of GAP with NFPEG3N3 increases the volume-specific impulse by 77 N s dm^−3^ and improves oxygen balance relative to Bu-NENA, identifying NFPEG3N3 as a competitive energetic plasticizer where volume efficiency is critical. These results justify advancing NFPEG3N3 to cured-formulation studies that avoid direct contact with untreated ADN or apply mitigation strategies (*e.g.*, polymer coated ADN prills), alongside extended HFMC and aging at multiple temperatures and mechanical property evaluation. Overall, NFPEG3N3 offers a favorable balance of compatibility with key components and performance potential, warranting further development for GAP-based PBX and propellant applications under appropriately controlled component selections.

## Experimental

### Materials

Desmodur N100, a commercial trifunctional isocyanate hardener for polyurethane coating systems, was obtained from Covestro Deutschland AG, Germany. GAP diol (charge 06S15) was purchased from Eurenco, France. HMX Grade B was obtained from Eurenco Bofors AB, Sweden. Prills of ADN (charge P95) were obtained from the Swedish Defense Research Agency (FOI).^22^

### Synthesis

NFPEG3N3 was synthesized at Fraunhofer ICT in accordance with the literature.^16^

### Characterization methods

Dynamic thermogravimetry (TG) was performed under a nitrogen flow of 25 mL min^−1^ using a Discovery 5500 TG system from TA Instruments (subsidiary of Waters Corporation, USA) and platinum pans. The samples were quickly heated to 50 °C at a rate of 10 °C min^−1^, and then to 500 °C at a rate of 2 °C min^−1^. Samples of 1 mg were measured for pure substances. Mixtures (1 : 1 ratio by weight) were measured as 2 mg samples. Heat-flow microcalorimetry (HFMC) was performed using a TAM (Thermal Activity Monitor) Type III from TA Instruments (subsidiary of Waters Corporation, USA). The instrument was originally developed by Thermometric AB, Sweden.^27^ Samples of 1 g were filled into a glass vial, which was placed inside an air-filled 4 mL stainless-steel ampoule. The steel ampoule was closed tightly and placed into the measuring device, which was submerged in an oil bath under isothermal conditions. The measurements were conducted at 80 °C for a 15-day test period.

### Thermodynamic calculations

The heat of explosion of the investigated mixtures was calculated with the ICT thermodynamic code in constant-volume mode.^28^ The loading density was set to *Δ* = 0.1, and the temperature freeze-out threshold for the water–gas equilibrium was set to *T* = 1500 K. As water is present primarily as vapor at the testing temperature of 80 °C, it was assumed to be in its gaseous state in the calculations. The performance of composite propellant formulations was calculated with the ICT thermodynamic code in constant-pressure mode using an expansion ratio of 70 : 1 and frozen-equilibrium values. The thermochemical parameters of GAP diol, HMX, ADN, and dioctyl adipate (DOA) were obtained from the ICT thermodynamic code's internal database.

## Conflicts of interest

There are no conflicts to declare.

## Supplementary Material

RA-015-D5RA05154A-s001

## Data Availability

The data supporting this article have been included as part of the supplementary information (SI). Supplementary information: molecular structures, dynamic TG curves, tabulated results of the HFMC compatibility test, tabulated values for the specific impulses, and SEM images of ADN P95 have been included as part of the SI (S1–S6). See DOI: https://doi.org/10.1039/d5ra05154a.
