# Supplementary material for: Energetic plasticizers for GAP-based formulations with ADN: compatibility and performance evaluation of a nitrofurazanyl ether
Source: RSC Adv. 2025 Oct 6;15(44):37006–11. doi: 10.1039/d5ra05154a (PMC12498327; doi:10.1039/d5ra05154a)
Supplement: RA-015-D5RA05154A-s001 [file RA-015-D5RA05154A-s001.pdf]

This journal is © The Royal Society of Chemistry 2025

Supplementary Information

S1 Molecular structures of representative energetic plasticizers used as reference compounds.

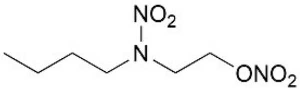

Bu-NENA
N-butyl-N-nitratoethylnitramin

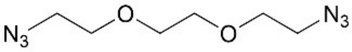

BATEG
1,2-bis(2-azidoethoxy)ethane

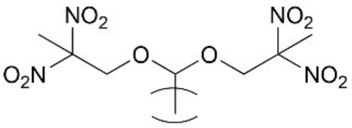

BDNPA/F
bis(2,2-dinitropropyl)acetal/ formal

S2 Repetitive units of the prepolymers GAP diol and HTPB.

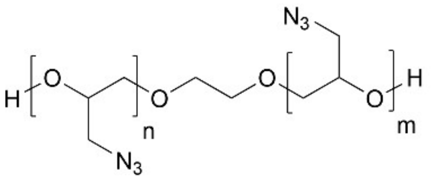

GAP diol
glycidyl azido polymer
diol initiator: ethan-1,2-diol

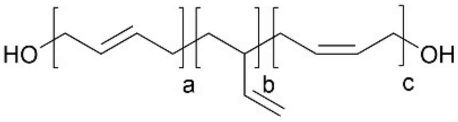

HTPB
hydroxyl-terminated polybutadiene

S3 Scanning electron microscope images of the used spherical prills of ADN.

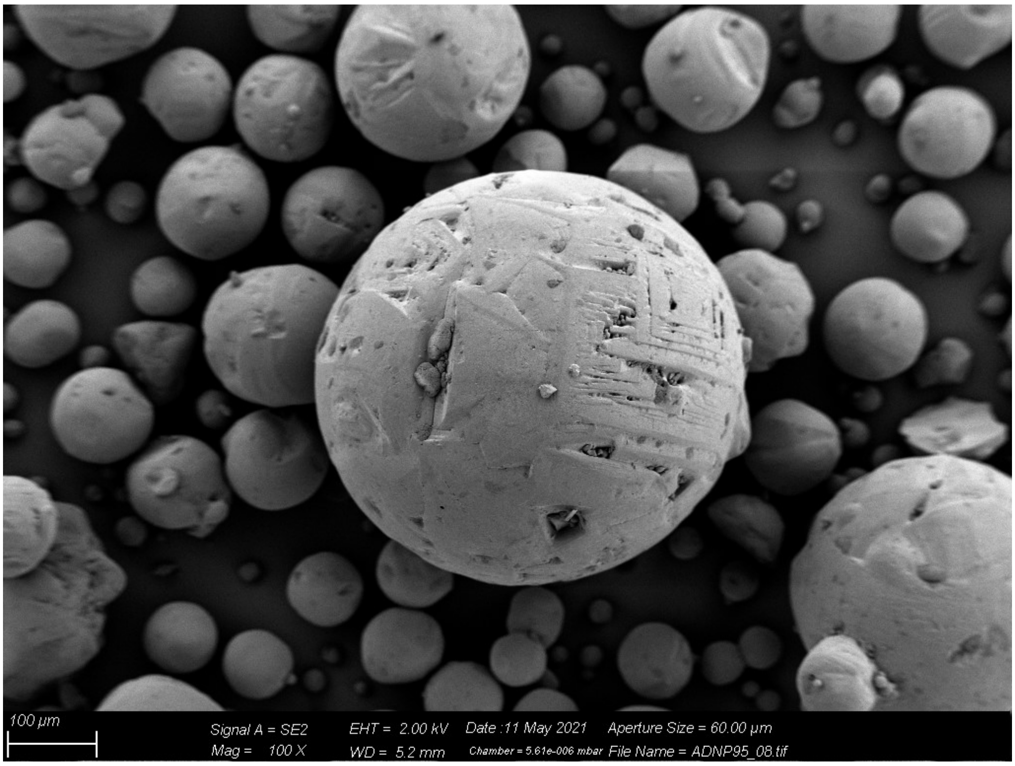
100 µm
Signal A = SE2
Mag = 100 X
EHT = 2.00 kV
WD = 5.2 mm
Date :11 May 2021
Chamber = 5.61e-006 mbar
Aperture Size = 60.00 µm
File Name = ADNP95_08.tif

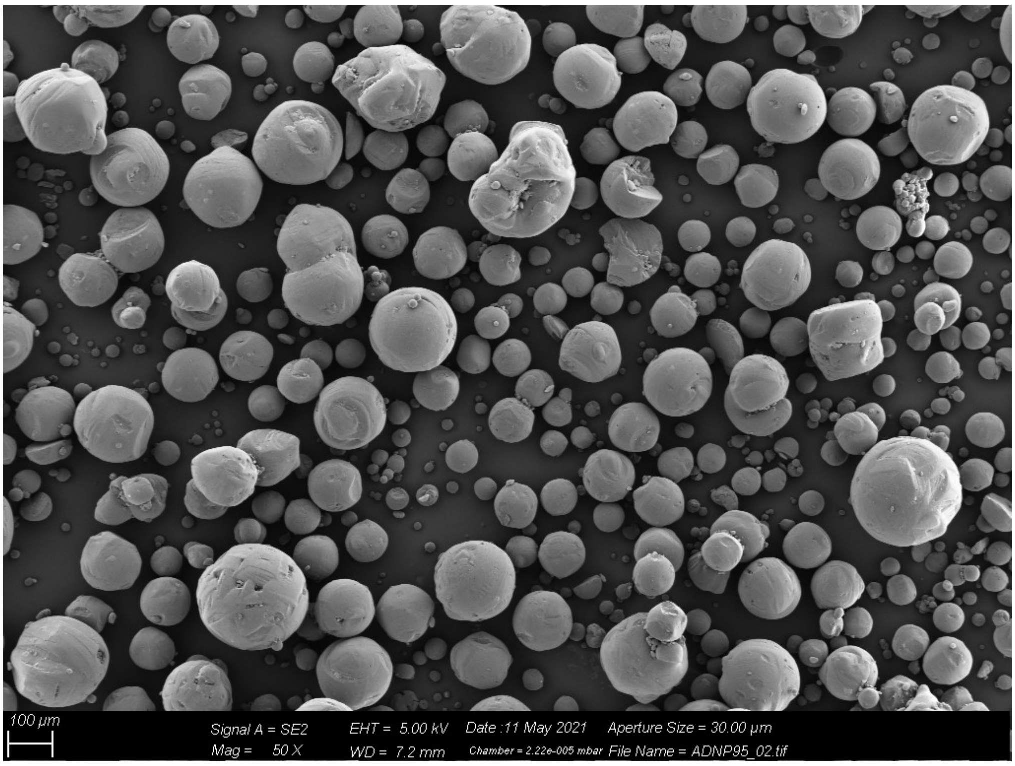
100 µm
Signal A = SE2
Mag = 50 X
EHT = 5.00 kV
WD = 7.2 mm
Date :11 May 2021
Chamber = 2.22e-005 mbar
Aperture Size = 30.00 µm
File Name = ADNP95_02.tif

S4 Dynamic thermogravimetry (TG) curves.

Pure compounds

NFPEG3N3, liquid

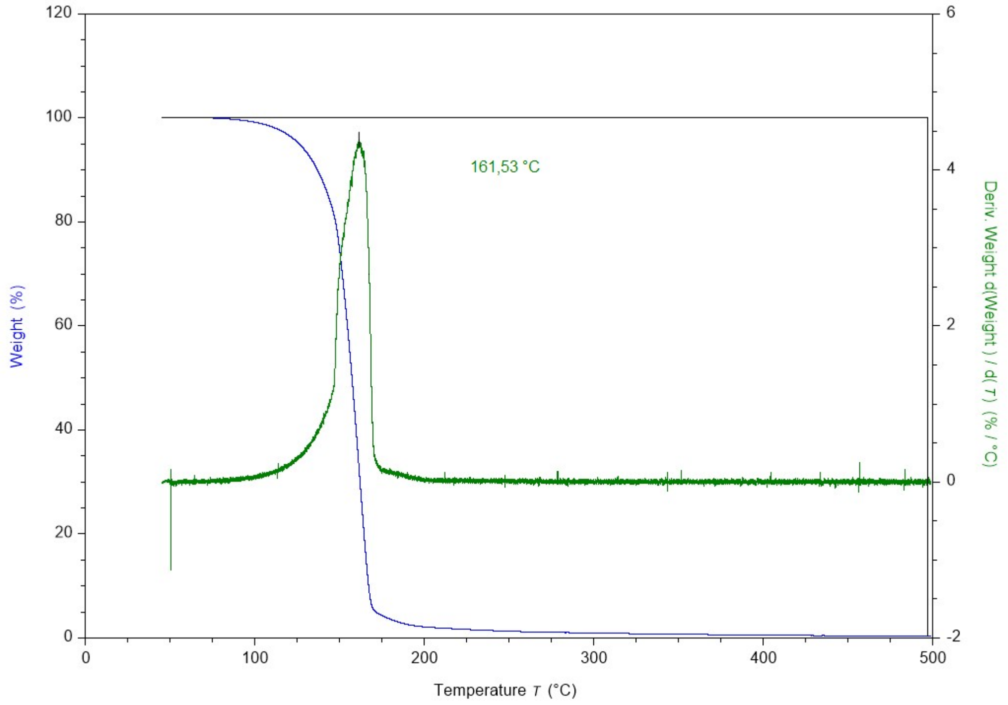

HMX, solid

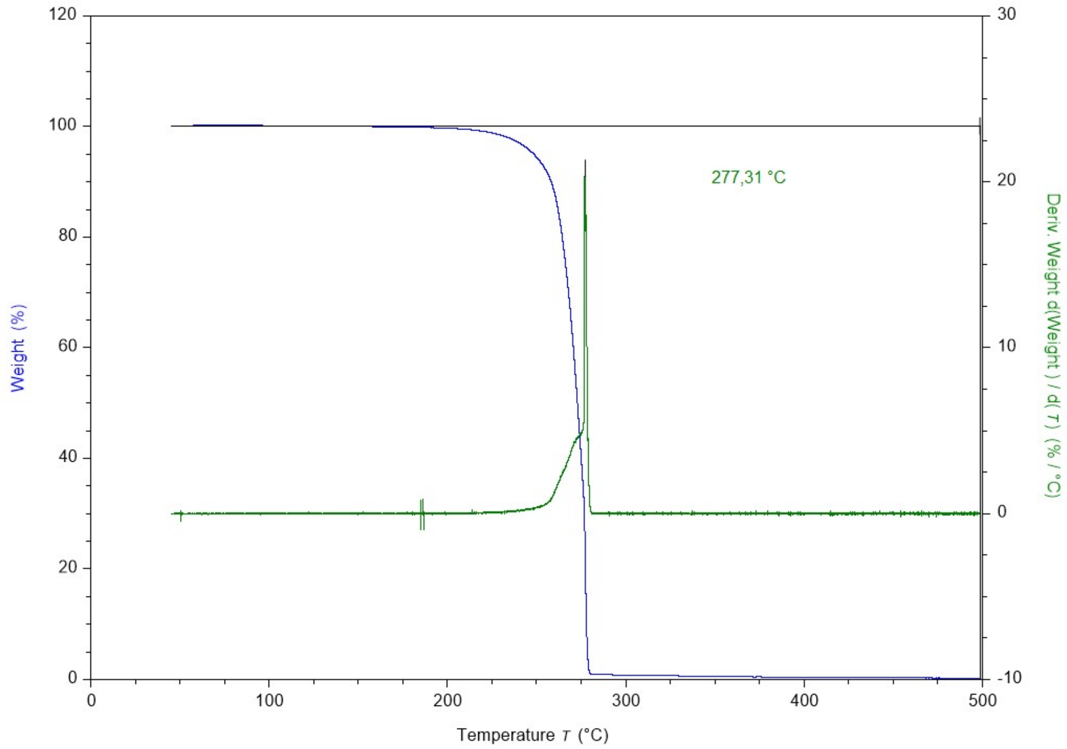

GAP diol, liquid

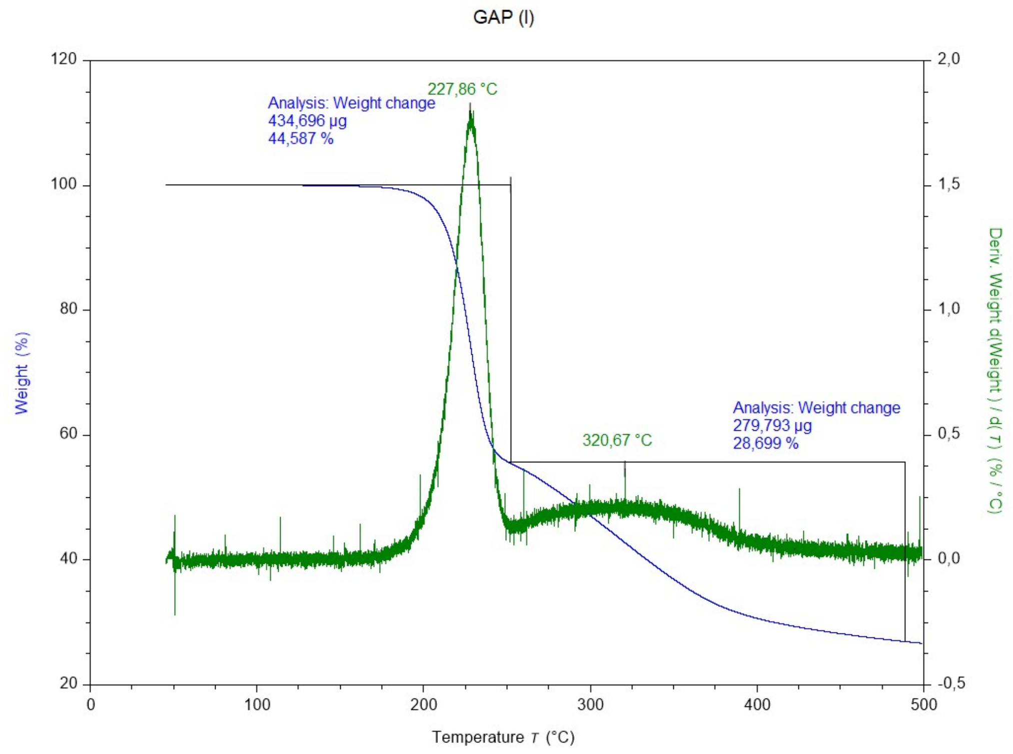

Desmodur N100, liquid

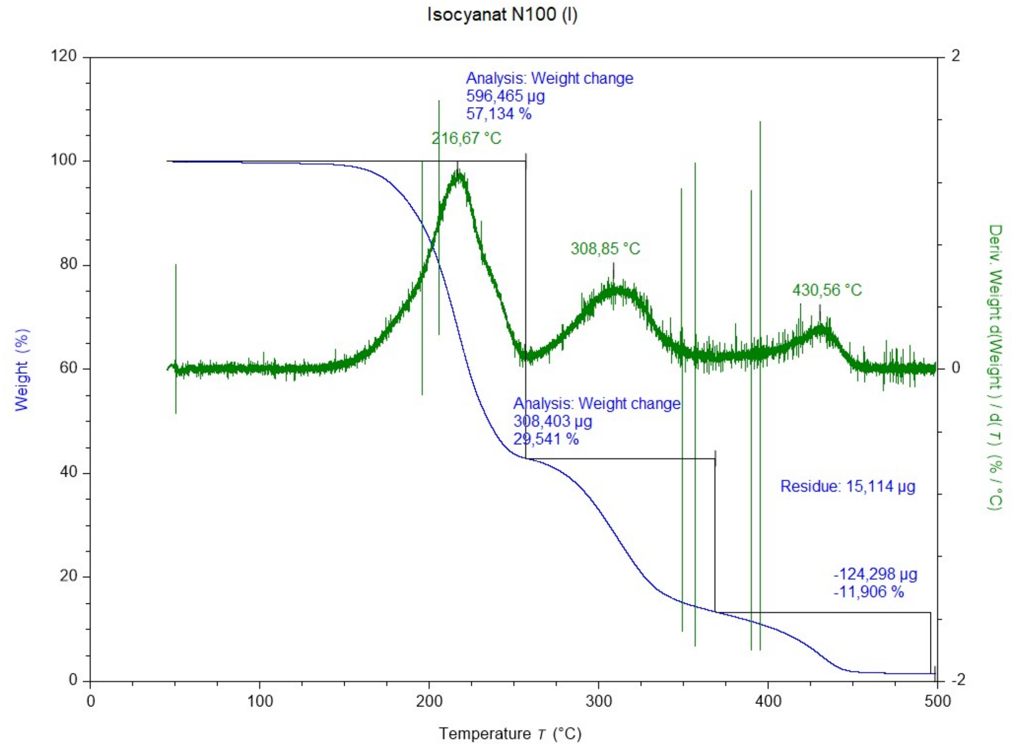

Ammonium dinitramide (ADN), solid

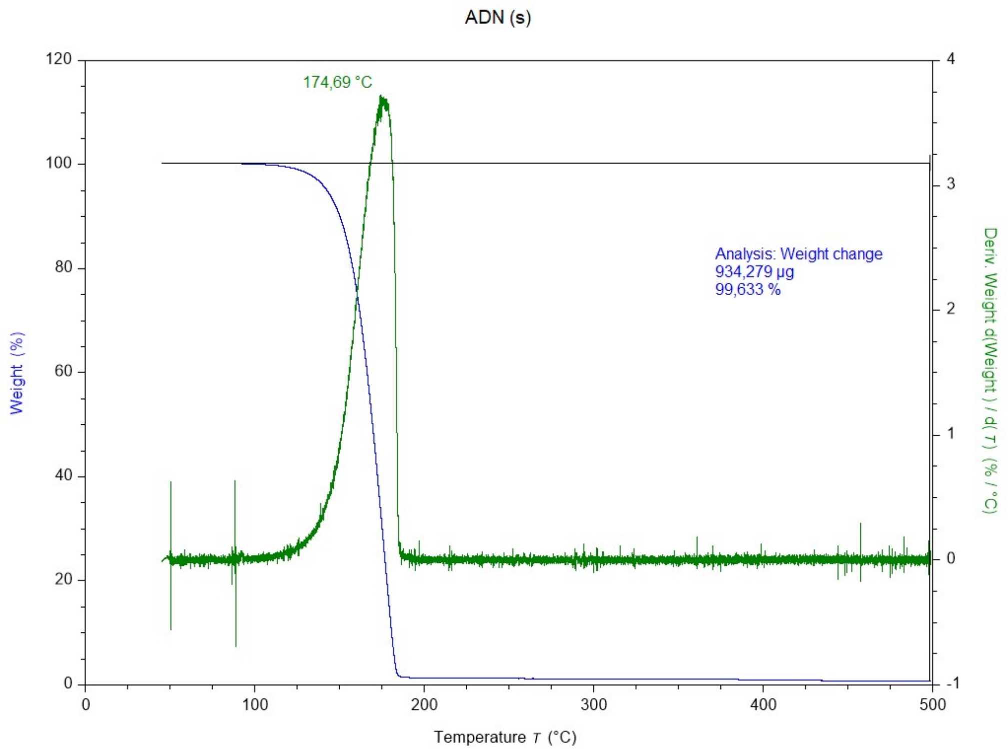

Mixtures

HMX-NFPEG3N3

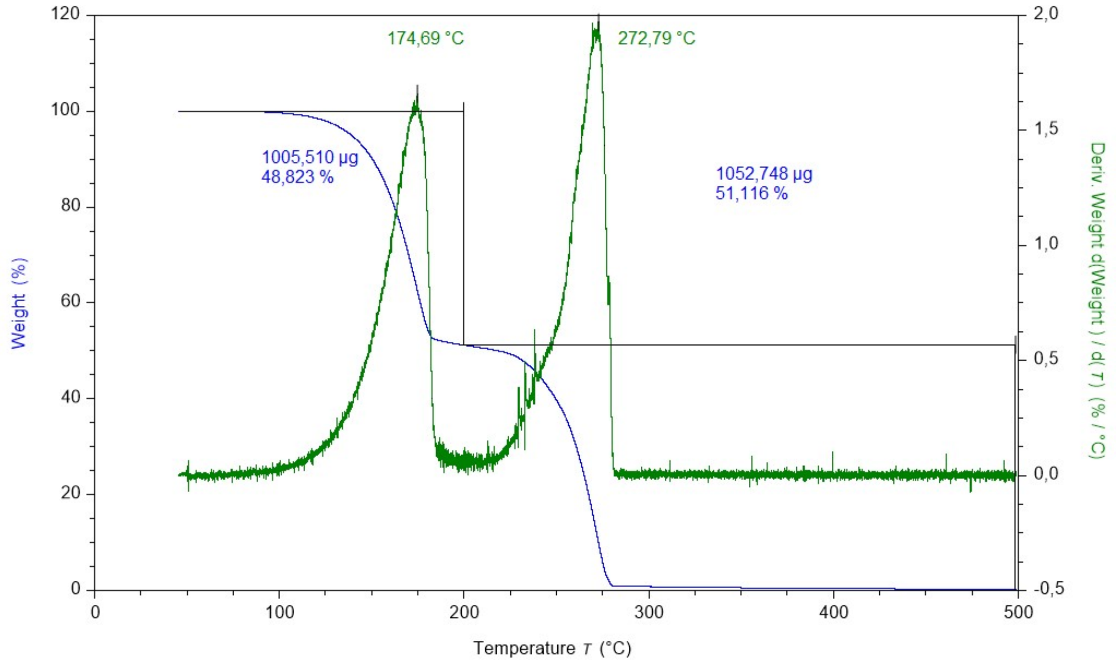

GAP diol-NFPEG3N3

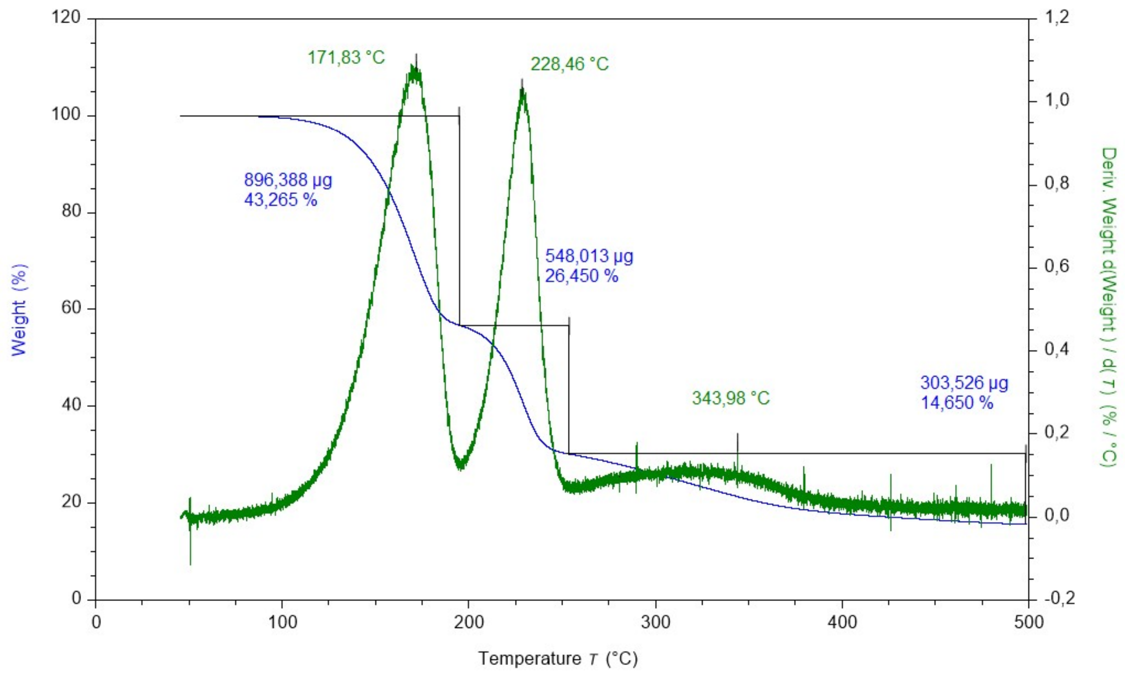

Desmodur N100-NFPEG3N

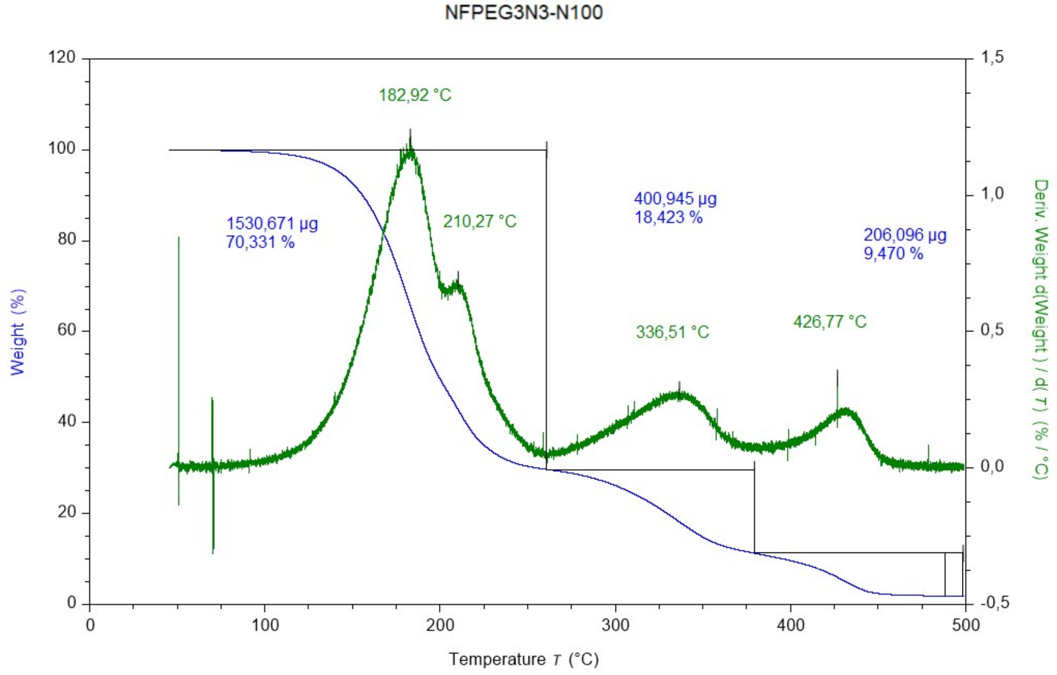

ADN-NFPEG3N3

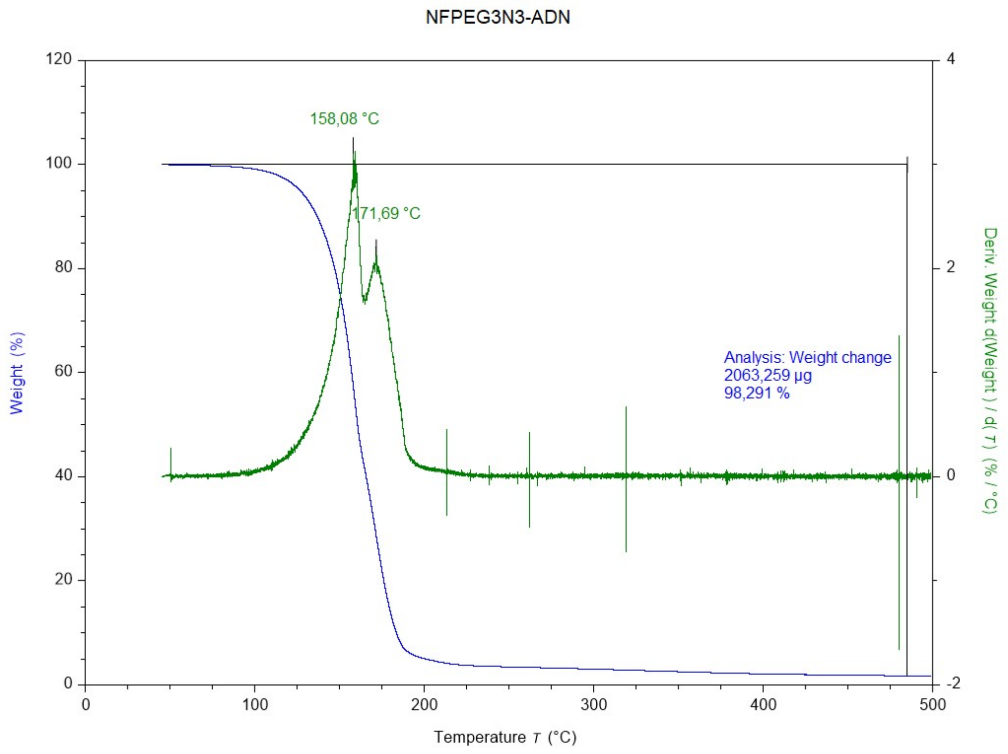

S5 Heat flow and reactivity data of the HFMC compatibility test.

Compound	$Q_{Ex,M}$ [J/g]	1% $Q_{Ex,M}$ [J/g]	Q_C [J/g]	$Q_{NFPEG3N3}$ [J/g]	$Q_{M,calc}$ [J/g]	Q_M [J/g]	Q_R [J/g]	Assmt.
HMX	3842	38.42	-0.03	19.59	9,78	10.57	0.79	ff.
GAP diol	3225	32.25	3.47		11.53	13.77	2.24	ff.
Desmodur® N100	-	30.00*	10.20		14,90	18.57	3.67	ff.
ADN	4190	41.90	3.22		11,41	35.87	24.46	ff.

S6 Mass and volume specific impulse data of composite propellant formulations (70% filler, 30% binder: GAP + plasticizer) calculated with the ICT thermodynamic code.

Plasticizer content [wt.%]	Mass spec. imp. with NFPEG3N3 [N s/Kg]	Mass spec. imp. with BDNPAF [N s/Kg]	Mass spec. imp. with Bu-NENA [N s/Kg]	Mass spec. imp. with BATEG [N s/kg]
0	2456	2456	2456	2456
5	2467	2475	2469	2457
10	2477	2490	2481	2458
15	2486	2499	2493	2459
20	2493	2502	2504	2460

Plasticizer content [wt.%]	Volume spec. imp. with NFPEG3N3 [N s/Kg]	Volume spec. imp. with BDNPAF [N s/Kg]	Volume spec. imp. with Bu-NENA [N s/Kg]	Volume spec. imp. with BATEG [N s/kg]
0	3989	3989	3989	3989
5	4017	4038	3996	3961
10	4043	4081	4001	3933
15	4066	4114	4006	3905
20	4088	4139	4009	3877
